# Relief of chronic pain associated with increase in midline frontal theta power

**DOI:** 10.1097/PR9.0000000000001040

**Published:** 2022-10-10

**Authors:** Nabi Rustamov, Elizabeth A. Wilson, Alexandra E. Fogarty, Lara W. Crock, Eric C. Leuthardt, Simon Haroutounian

**Affiliations:** a Department of Neurological Surgery, Washington University School of Medicine, St. Louis, MO, USA; b Center for Innovation in Neuroscience and Technology, Division of Neurotechnology, Washington University School of Medicine, St. Louis, MO, USA; c Department of Anesthesiology, Washington University School of Medicine, St. Louis, MO, USA; d Department of Orthopedic Surgery, Division of Physical Medicine & Rehabilitation, Washington University School of Medicine, St. Louis, MO, USA; e Washington University Pain Center, St. Louis, MO, USA; f Department of Biomedical Engineering, Washington University in St. Louis, Louis, MO, USA; g Department of Neuroscience, Washington University School of Medicine, St. Louis, MO, USA; h Department of Mechanical Engineering and Materials Science, Washington University in St. Louis, St. Louis, MO, USA

**Keywords:** Chronic pain, Experimental acute pain, Nerve block, Pain relief, Midline frontal theta power

## Abstract

**Introduction:**

There is a need to identify objective cortical electrophysiological correlates for pain relief that could potentially contribute to a better pain management. However, the field of developing brain biomarkers for pain relief is still largely underexplored.

**Objectives:**

The objective of this study was to investigate cortical electrophysiological correlates associated with relief from chronic pain. Those features of pain relief could serve as potential targets for novel therapeutic interventions to treat pain.

**Methods:**

In 12 patients with chronic pain in the upper or lower extremity undergoing a clinically indicated nerve block procedure, brain activity was recorded by means of electroencephalogram before and 30 minutes after the nerve block procedure. To determine the specific cortical electrophysiological correlates of relief from chronic pain, 12 healthy participants undergoing cold-pressor test to induce experimental acute pain were used as a control group. The data were analyzed to characterize power spectral density patterns of pain relief and identify their source generators at cortical level.

**Results:**

Chronic pain relief was associated with significant delta, theta, and alpha power increase at the frontal area. However, only midfrontal theta power increase showed significant positive correlation with magnitude of reduction in pain intensity. The sources of theta power rebound were located in the left dorsolateral prefrontal cortex (DLPFC) and midline frontal cortex. Furthermore, theta power increase in the midline frontal cortex was significantly higher with chronic vs acute pain relief.

**Conclusion:**

These findings may provide basis for targeting chronic pain relief via modulation of the midline frontal theta oscillations.

## Abstract 2

Unique electroencephalogram signatures of relief from chronic pain demonstrate theta power increase in the midline frontal cortex.

## 1. Introduction

Currently available clinical tools to evaluate pain experience are reliant on subjective reports, which are influenced by many factors, such as the cognitive state and mood.^23,26,68^ There is a need to identify objective features for pain and pain relief, ie, biomarkers related to physiological pain processing mechanisms, that could help to further characterize and hopefully better manage pain.^15,20,40,69^ Additionally, objective biomarkers of pain relief could also serve as potential targets for novel therapeutic interventions to treat pain. However, the field of developing brain biomarkers for pain, and equally as important, for pain relief, is still largely underexplored (see Ref. 71 for a review).

The systematic assessment of cortical oscillations is a promising approach for the investigation of brain activity patterns associated with chronic pain and its relief. However, few studies have addressed chronic pain and the results are not fully consistent (see Refs. 52 and 53 for a review). Chronic pain seems to be associated with abnormal oscillations at theta frequencies, although the specificity of these findings has remained unclear. Previous work has posited that these abnormal theta oscillations are the result of a thalamocortical dysrhythmia (TCD) because of cell-specific neural firing in the thalamus.^38,39,60^ It is unclear, however, how universal this model is across patients with chronic pain.^35,63^

Although there are preliminary studies on cerebral processing of acute and chronic pain,^53^ evidence about the neurophysiological encoding of pain relief is scarce. There are a *few studies* that have explored recovery period after thermal painful stimulation, which reported over-recovery in theta and alpha power compared with resting baseline.^8^ Additionally, our previous work has shown the association between recovery from acute thermally induced pain and prefrontal theta power rebound.^58^ Although experimental tonic pain is thought to closely simulate the subjective properties of clinical chronic pain because its high level of unpleasantness,^28,44,45,55^ those results cannot be necessarily extrapolated to chronic pain relief. Although tonic pain has only been explored with painful thermal tests that last minutes, chronic pain in the clinical practice is not restricted to a single etiology and occurs over much longer periods. Thus, the objective of the current study was to investigate cortical electrophysiological correlates associated with chronic pain relief. Determining cortical activity patterns that are implicated in chronic pain relief would be a crucial step in evaluating their potential as a clinically relevant biomarker of pain relief. To that end, brain activity was recorded by means of electroencephalogram (EEG) in patients with chronic upper or lower extremity pain before and 30 minutes after a clinically indicated diagnostic or therapeutic nerve block procedure. Most of the patients in this setting would be expected to obtain some pain relief from the procedure, and this design was selected to avoid unnecessary risks of performing interventional procedures like nerve blocks for the study purposes outside standard of care. We hypothesized that although relief from chronic and acute pain will share substantial neural dynamics, because of the maladaptive nature of chronic pain, relief from chronic pain will have additional distinct features.

## 2. Materials and methods

### 2.1. Ethical approval

This study was approved by the institutional review board of Washington University School of Medicine in St. Louis. All experimental procedures conformed to the standards set by the latest revision of the Declaration of Helsinki. All participants provided written informed consent before participation in the study.

### 2.2. Participants

#### 2.1.1. Chronic pain group

Patients were recruited from the Washington University Pain Management Center. Inclusion criteria were patients of any age between 18 and 70 years with chronic pain (pain for at least 3 months) planned to undergo clinically indicated nerve block procedure. Exclusion criteria included (1) lack of written informed consent; (2) the presence of *other* pain symptoms with pain severity >4 on a 0 to 10 numerical rating scale (NRS) on the day of the nerve block procedure; (3) pain exacerbations in the past 2 weeks requiring medication changes, procedures, or hospitalization; (4) a major neurological condition known to produce changes in the oscillatory cortical activity. All patients maintained their normal medication regimen at the time of testing.

#### 2.1.2. Control group (experimental acute pain)

Healthy participants aged between 18 and 70 years, with no major conditions of any organ system were invited to participate in the study. This cohort of healthy volunteers has been described previously.^58^

### 2.2. Study design

After screening and obtaining written informed consent, participants were asked to complete questionnaires that included basic demographic information, Hospital Anxiety and Depression Scale, and Pain Catastrophizing Scale.^3,32,70,74^ Participants were seated on a chair and asked to limit their movements to minimal throughout the EEG recording. They were instructed to fixate on a fixation cross presented centrally on the screen to avoid excessive eye blinking while keeping their eyes open.

#### 2.2.1. Chronic pain group

**Figure 1.**
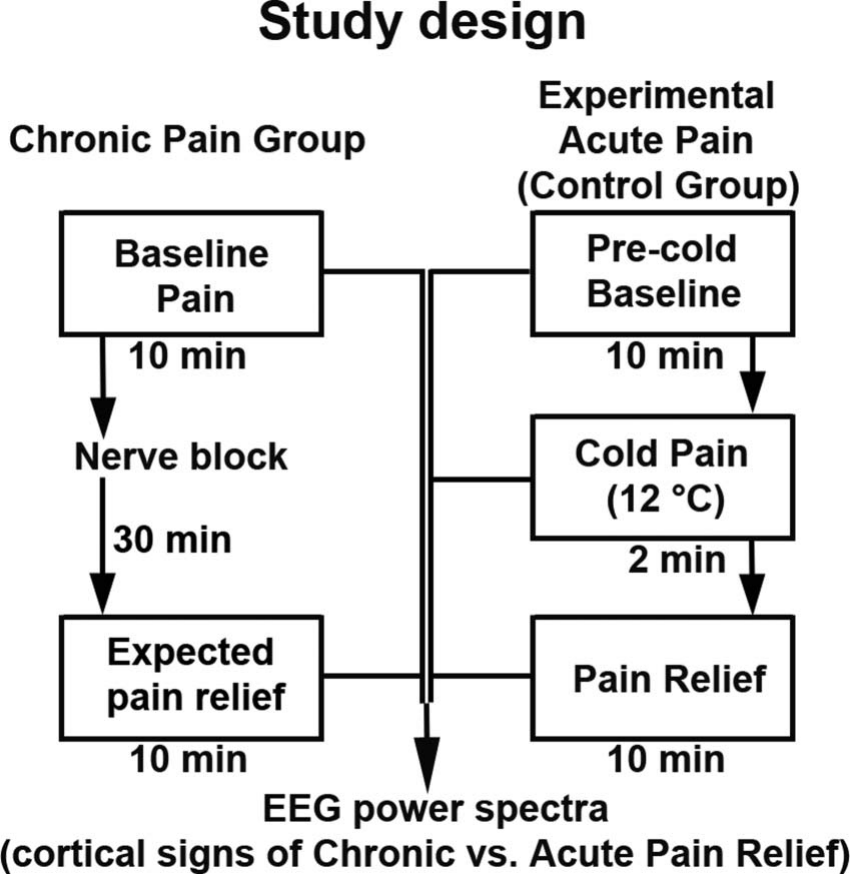
Experimental paradigm. Left panels: Chronic pain group. Patients with chronic pain underwent a clinically indicated nerve block procedure, and 10-minute EEG data were collected before and 30 minutes after the nerve block. Right panels: Experimental acute pain (control group). Healthy participants underwent a single session of EEG recording that included 3 consecutive conditions: precold baseline (10 minutes), cold pain (2 minutes), and pain relief (10 minutes). Electroencephalogram data were analyzed with respect to oscillatory brain activity followed by source estimation of power spectral density. EEG, electroencephalogram.

In patients with chronic pain undergoing a clinically indicated nerve block procedure, EEG was recorded during 2 consecutive conditions: baseline (with ongoing pain) and 30 minutes after the nerve block (Fig. 1, left panels). Electroencephalogram data were 10 minutes per condition. Patients rated their pain intensity on a NRS ranging from no pain to the worst tolerable pain (0–10).

#### 2.2.2. Control group (experimental acute pain)

In healthy participants, EEG was recorded during baseline, cold pain, and pain relief conditions (Fig. 1, right panels). Baseline EEG data were collected for 10 minutes. Then, thermostat-controlled circulating cold-water bath was used to induce moderate pain (see Ref. 58 for the cold-pressor test procedure). Participants rated cold pain intensity on NRS, with 0 being no pain and 100 being the worst tolerable pain. Another 10-minute EEG data were collected immediately after the cold-pressor test. Participants reported in case they had pain at the end of the pain relief period.

### 2.3. Electroencephalogram recording and processing

The EEG was recorded by means of 24-channel amplifier, using wireless dry electrodes that were mounted on the EEG headset in an International 10 to 20 System montage (DSI 24; Wearable Sensing, San Diego, CA; see Ref. 58 for the EEG data recording parameters). The raw EEG data were preprocessed in MATLAB environment (Mathworks, Natick, MA; see Refs. 56 57,58 for the EEG data processing pipeline). Frequency bands were specified in the following manner: 1 to 3 Hz: delta; 4 to 7 Hz: theta; 8 to 13 Hz: alpha; 14 to 29 Hz: beta; 30 to 58 Hz: low gamma; 62 to 100 Hz: high gamma.^53^

### 2.4. Power spectral density

The power spectral density (PSD) was computed for all the conditions using Welch method^73^ as described previously (see Refs. 56 and 58 for a data processing pipeline). The power spectrum from 1 to 100 Hz was obtained. The average PSD values across participants were normalized relative to baseline.

### 2.5. Source estimation of power change

A source localization of power change was performed to estimate the cortical sources associated with pain relief (see Refs. 58 and 59 for a source estimation pipeline). The power decomposition on the source from 4 to 7 Hz was computed using Welch method.^73^ The averaged data across participants were normalized relative to baseline.

### 2.6. Statistical analyses

This study relied on a between-group design to compare electrophysiological features associated with chronic pain relief (chronic pain group) with those of experimental acute pain relief in healthy participants (control group). All results are expressed as mean ± SD unless specified. We used nonparametric permutation statistics that makes assumptions without regard to any underlying distribution^31,41,48^ (see Ref. 58 for a statistical procedure). All statistical tests were two-tailed with significance thresholds set to *P* ≤ 0.05. The *P*-values were adjusted using a false discovery rate procedure to control for multiple comparisons.

Correlation analysis was conducted between changes in EEG power spectra and pain intensity after the nerve block procedure. We used a nonparametric Spearman rank correlation that allows to measure nonlinear relation between 2 random variables.^27,56,61^ Correlations were computed for the statistically significant PSD effects (theta power increase at the F3 and Fz electrodes; delta and alpha power increase at the Fz electrode). The PSD values for the post-block condition relative to baseline pain were averaged across a frequency band obtaining a single value per electrode, participant. The percentage differences in pain ratings between baseline and after the nerve block were computed. Correlations were computed by comparing changes in PSD values and pain intensity ratings after the nerve block.

Twelve participants were selected for the nerve block study as a convenience sample to detect a significant change in pain intensity from 6 (±3) to 3 (±3) on 0 to 10 NRS, with 90% power and α = 0.05 in a paired *t* test (G*Power 3.1, Dusseldorf, Germany).

## 3. Results

### 3.1. Participant characteristics

**Figure 2.**
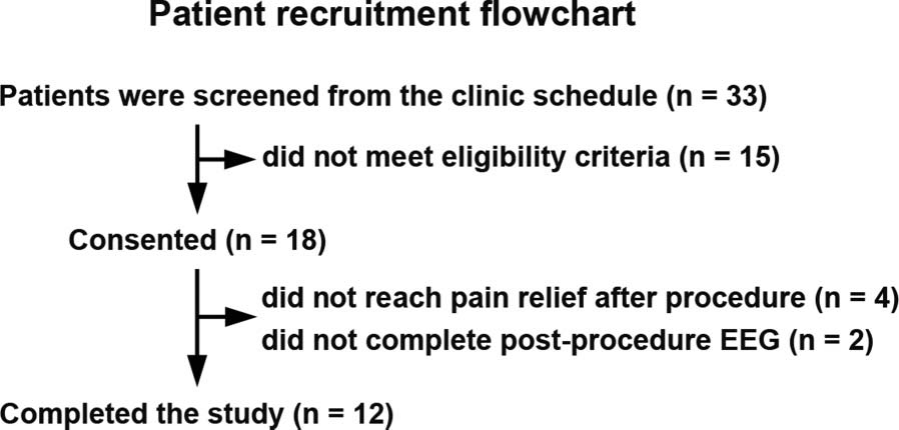
Patient recruitment flowchart. Thirty-three patients with chronic pain were screened from the clinic schedule. Fifteen patients did not meet the eligibility criteria. Eighteen patients consented to participate in the study. Twelve patients completed the study.

The patient recruitment flowchart is outlined in Figure 2. The demographics and baseline pain characteristics of the cohort are provided in Table 1. A total of 12 patients completed the study, of which 11 were female. The mean age was 45.1 ± 12.0 years (age range: 31–66 years). Patients had pain for an average of 2.1 ± 1.5 years, for which they reported taking 4.7 ± 1.7 analgesic medications. On average, patients reported a baseline pain intensity of 6.1 ± 1.6 on a 0 to 10 NRS, pain catastrophizing scale score of 28.0 ± 13.5, hospital anxiety score of 9.5 ± 4.9, and hospital depression score of 7.5 ± 4.1, indicating mild anxiety and depression. Patient characteristics, including comorbidities, home medications, and details of the nerve block procedure, are provided in Table 2.

**Table 1:** Patient demographics and baseline pain characteristics.

	Mean (±SD)
Age	45.1 ± 12.0
Female/male sex	N = 11/1
Pain duration	2.1 ± 1.5
Pain intensity per 0–10 NRS	6.1 ± 1.6
Chronic conditions	2.6 ± 2.4
Analgesic medications	4.7 ± 1.7
HADS total score: Anxiety	9.5 ± 4.9
HADS total score: Depression	7.5 ± 4.1
Pain catastrophizing scale	28.0 ± 13.5
Rumination	10.3 ± 5.1
Magnification	5.5 ± 3.9
Helplessness	12.2 ± 5.8

HADS, Hospital Anxiety and Depression Scale; N, number; NRS, numerical rating scale.

**Table 2:** Patient characteristics.

	Sex, age	Index chronic pain condition	Duration of chronic pain (y)	Comorbid painful conditions	Comorbid medical diagnoses	Analgesic medications	Intervention
1	F, 36	CRPS of left upper extremity	1.0	None	None	Pregabalin, amitriptyline, baclofen, oxycodone	Left stellate ganglion block
2	F, 57	CRPS of right upper extremity	0.5	Thoracic outlet syndrome	Endometriosis, bipolar 1, asthma, GERD, panic disorder	Naltrexone, cannabidiol, cyclobenzaprine, depakote, gabapentin, nortriptyline	Right stellate ganglion block
3	F, 33	Lumbar radiculopathy with right lower extremity pain	3.5	Sacroiliac joint dysfunction Postlaminectomy syndrome	None	Acetaminophen, celecoxib, cyclobenzaprine, duloxetine, ibuprofen, tizanidine, tramadol	Lumbar transforaminal epidural steroid injection at right S1
4	F, 66	Lumbar radiculopathy with left lower extremity pain	5.0	Lumbar spondylosis	Asthma, diabetes, hypertension, incontinence, Depression	Cyclobenzaprine, pregabalin	Lumbar transforaminal epidural steroid injection at left L5
5	F, 45	CRPS of left lower extremity	3.6	None	None	Alpha lipoic acid, tizanidine, tramadol, turmeric	Left lumbar sympathetic block
6	M, 37	CRPS of left upper extremity	1.0	Phantom limb pain	None	Diclofenac, nortriptyline, sertraline, pregabalin, amitriptyline	Left stellate ganglion block
7	F, 47	CRPS of left lower extremity	3.1	Migraine Knee osteoarthritis	Adhesive capsulitis, hypertension, GERD	Baclofen, cyclobenzaprine, gabapentin, ibuprofen	Left lumbar sympathetic block
8	F, 39	CRPS of right upper extremity	1.7	None	PCOS, hypertension, asthma, diabetes, depression	Meloxicam, naltrexone, pregabalin	Stellate ganglion block
9	F, 31	CRPS of right upper extremity	0.9	None	Depression	Acetaminophen, cyclobenzaprine, ibuprofen, methocarbamol, pregabalin, naltrexone	Stellate ganglion block
10	F, 42	Facet arthropathy with axial low back pain	3.1	Cervical radiculopathy Migraine Sacroiliac joint dysfunction	GAD, hypothyroidism	Gabapentin	Local anesthetic medial branch nerve blocks to facet joints at lumbar levels, left L3, L4, L5, and S1
11	F, 63	Lumbar radiculopathy of with bilateral lower extremity radiation	1.9	Sacroiliac joint dysfunction Postlaminectomy syndrome Lumbar radiculopathy	Hypothyroidism, GERD, hyperlipidemia	Celecoxib, cyclobenzaprine, gabapentin	Lumbar transforaminal epidural steroid injection (selective nerve root injection) at right L4
12	F, 35	CRPS of right lower extremity	0.4	Hip osteoarthritis	Anxiety, hypertension	Diclofenac, duloxetine DR, mirtazapine, naltrexone, pregabalin	Lumbar sympathetic block

CRPS, complex regional pain syndrome; F/M, female and male.

Twelve healthy participants (6 women and 6 men; age: 29.7 ± 5.7 years; age range: 20–38 years; hospital anxiety score: 4.0 ± 2.4; hospital depression score: 1.4 ± 2.2) undergoing cold-pressor test to induce experimental acute pain served as a control group.

### 3.2. Pain ratings

#### 3.2.1. Chronic pain group

After the nerve block procedure, all patients reported a reduction in NRS pain score. Patients achieved a mean decrease of 58.3% ± 24.6% in pain level. This implies clinically meaningful average pain relief, surpassing the minimum clinically important difference threshold of 30% reduction in pain intensity.^42,47^ Overall, 10 of the 12 patients reached the minimum clinically important difference.

#### 3.2.2. Control group (experimental acute pain)

The average intensity of pain during the cold-pressor test was rated as 59.6 ± 11.0 on a 0 to 100 NRS. Individual pain ratings ranged from 45 to 80, mostly falling within the range of moderate pain.^34^ All participants reported pain intensity of zero by the end of the pain relief condition.

### 3.3. Changes

#### 3.3.1. Power spectral density

##### 3.3.1.1. Chronic pain group

**Figure 3.**
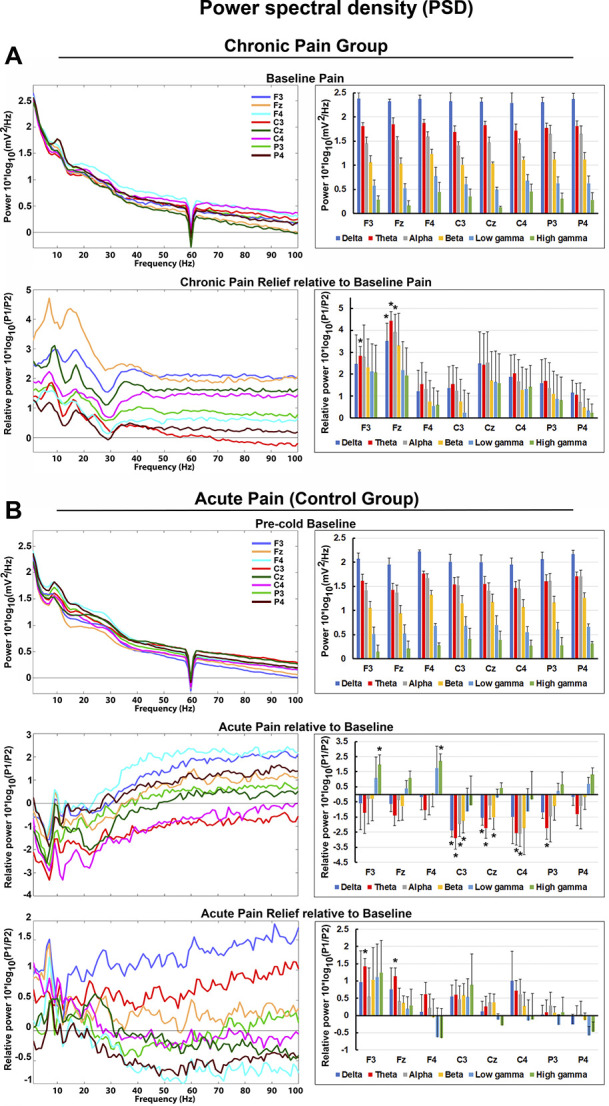
Power spectral density per group. Selection of frontoparietal electrodes with respect to regions of interest was presented. (A) Chronic pain group. Left panels: power spectra averaged across all patients. Log power spectra during baseline pain. Power spectra during chronic pain relief were normalized relative to baseline pain condition (log power ratio). Right panels: PSD values averaged across frequency bands. (B) Acute pain (control group). Left panels: power spectra averaged across all healthy controls. Log power spectra during precold baseline. Power spectra during acute pain and acute pain relief were normalized relative to precold baseline condition (log power ratio). Right panels: PSD values averaged across frequency bands. Electroencephalogram electrodes were depicted in 8 different colors. Frequency bands: delta (1–3 Hz), theta (4–7 Hz), alpha (8–13 Hz), beta (14–29 Hz), low gamma (30–58 Hz), and high gamma (62–100 Hz). PSD, power spectral density.

**Figure 4.**
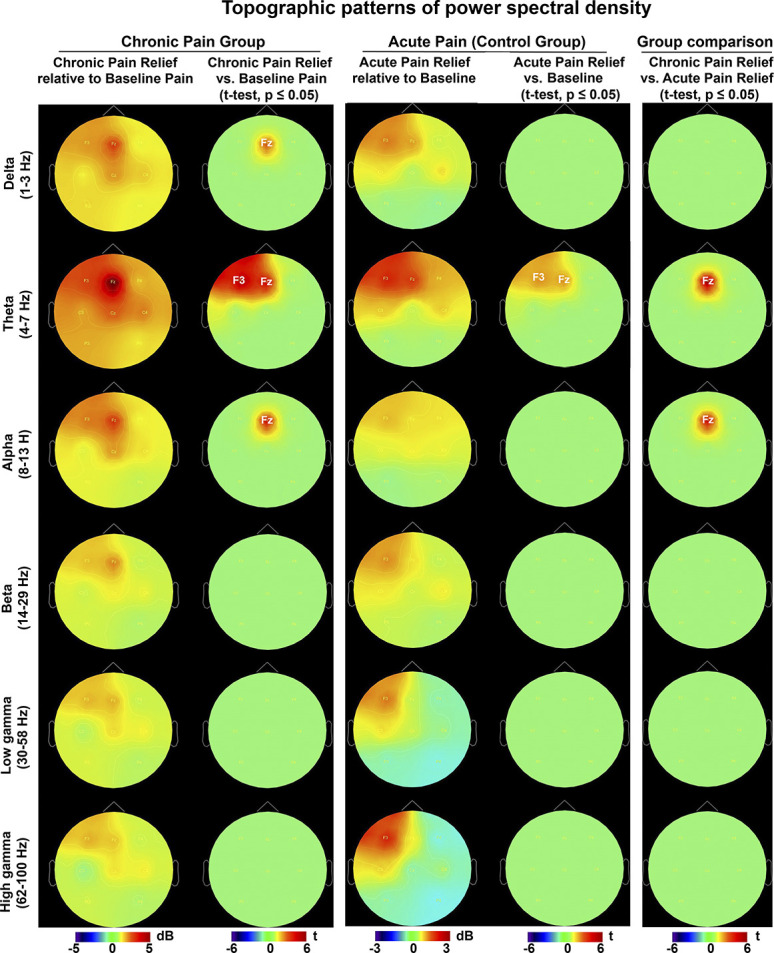
Topographic representation of power spectral density. Power spectra were normalized relative to baseline (dB = log power ratio). Positive and negative power changes are represented by red and blue colors, respectively. Electrode level t maps of the comparison between conditions as assessed by nonparametric permutation tests. Only electrodes whose t statistic exceeded a critical threshold of *P* ≤ 0.05 (two-tailed, FDR corrected) were retained. For the electrodes not showing significant effects, t values were set to zero. Left column: chronic pain relief. Middle column: acute pain relief (control group). Right column: between-group power comparison of chronic pain relief vs acute pain relief. FDR, false discovery rate.

**Figure 5.**
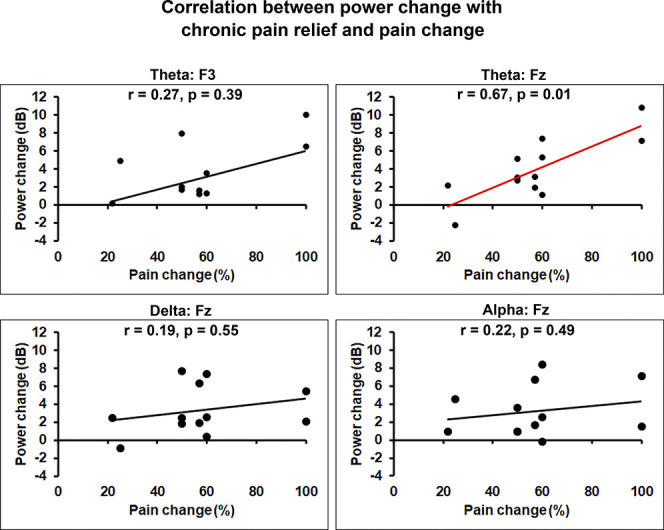
Relationships between power changes and chronic pain relief. Spearman rank correlations were run between changes in power spectra and pain intensity after the nerve block procedure. Correlations were assessed for the statistically significant power spectra effects (theta power increase at the F3 and Fz electrodes; delta and alpha power increase at the Fz electrode). Significance thresholds were set at *P* ≤ 0.05. Significant correlations between pain relief and theta power change at the Fz electrode were depicted by red color; *y*-axis, and *x*-axis, changes in power spectra and pain intensity following nerve block, respectively.

During chronic pain relief compared with baseline pain, theta power increased at the frontal area (significant effects at the F3 and Fz electrodes, both *P*s < 0.05, maximum effect at Fz, t = 5.0) (Fig. 3A, bottom panels, and Fig. 4 left panels). Similarly, delta and alpha frequency bands showed power increase at the midfrontal area (delta and alpha power: significant effects at the Fz electrode, both *P*s < 0.05, t = 3.5 and 4.0, respectively). Correlations between changes in power spectra and chronic pain intensity ratings with chronic pain relief are shown in Figure 5. Correlation coefficients between theta power change at the F3 and Fz electrodes and percentage change in pain score were 0.27 (*P* = 0.39) and 0.67 (*P* < 0.01), respectively. These results imply significant positive correlation between midfrontal theta power increase and reduction in pain intensity. Conversely, delta and alpha power increase at the midfrontal area correlated poorly with pain score change, with correlation coefficients of 0.19 (*P* = 0.55) and 0.22 (*P* = 0.49), respectively.

##### 3.3.1.2. Control group (experimental acute pain)

Compared with baseline, acute pain resulted in suppression of theta and alpha power over the central area bilaterally (theta power: significant effects at the C3, P3, Cz, and C4 electrodes, all *P*s < 0.05, maximum effect at C3, t = −4.9; alpha power: significant effects at the C3 and C4 electrodes, both *P*s < 0.05, maximum effect at C3, t = −2.6) (Fig. 3B, middle panels). In delta and beta frequency bands, power reduction effects were lateralized (delta and beta power: significant effects at the C3 and Cz electrodes, both *P*s < 0.05, maximum effects at C3, t = −3.5 and −2.6, respectively). High gamma frequency band showed power increase frontally (significant effects at the F3 and F4 electrodes, both *P*s < 0.05, maximum effect at F4, t = 3.1). Compared with baseline, acute pain relief was characterized by theta power increase over the frontal area (significant effects at the F3 and Fz electrodes, both *P*s < 0.05, maximum effect at F3, t = 3.2) (Fig. 3B, bottom panels, and Fig. 4 middle panels). Acute pain relief vs acute pain contrast revealed theta power rebound over the frontocentral area with lateralization to the left side (significant effects at the F3, Fz, C3, and Cz electrodes, all *P*s < 0.05, maximum effect at C3, t = 4.5) (Fig. 3B, bottom vs middle panels).

#### 3.3.2. Group differences in power spectral density

**Figure 6.**
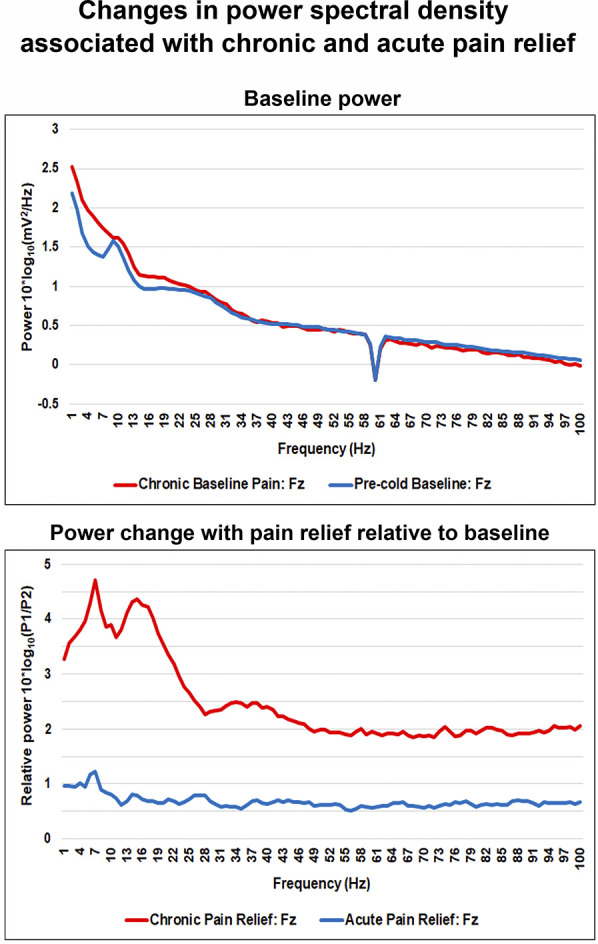
Changes in power spectral density at the Fz electrode with chronic and acute pain relief. Power spectra were averaged across participants per group. Top panel: baseline log power spectra at the Fz electrode. Bottom panel: power spectra at the Fz electrode during chronic and acute pain relief relative to baseline conditions (log power ratio).

We compared baseline EEG bandwidth activity between the groups as measured over all the electrode sites. A nonsignificant trend emerged for differences in baseline delta and theta power at the Fz electrode (delta and theta power: both *P*s > 0.05, t = 2.2 and t = 2.4, respectively) (Fig. 3A, B, top panels, and Fig. 6, top panel). This was in the expected direction of the original findings reporting increased theta power in chronic pain patients compared with healthy participants but did not reach significance.^62,67^ Between-group comparison of chronic pain relief vs acute pain relief yielded significant theta and alpha power increase at the midfrontal area (theta and alpha power: significant effects at the Fz electrode, both *P*s < 0.05, t = 4.5 and t = 4.2, respectively) (Fig. 4, right panels, and Fig. 6, bottom panel).

#### 3.3.3. Source estimation of theta power rebound with pain relief

**Figure 7.**
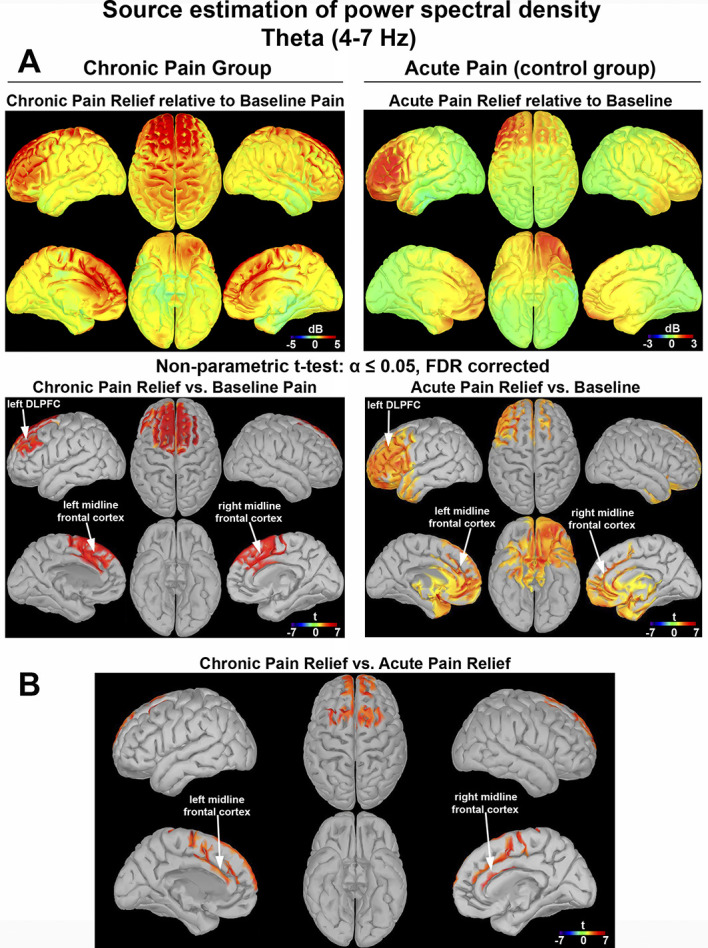
Source estimation of theta power rebound with chronic and acute pain relief. Theta oscillations on source level. Positive and negative relationships were depicted by red and blue colors, respectively. Source estimation was represented as t values, based on a voxelwise nonparametric permutation tests on power source space. Only voxels whose t statistic exceeded a critical threshold of *P* ≤ 0.05 (two-tailed, FDR corrected) were retained. For the voxels not showing significant effects, t values were set to zero. (A) Left column: chronic pain group. Right column: acute pain (control group). (B) Between-group power source comparison of chronic pain relief vs acute pain relief. DLPFC, dorsolateral prefrontal cortex; FDR, false discovery rate.

Theta power increase after the nerve block showed significant correlation with chronic pain relief. Moreover, theta power was significantly higher during acute pain relief compared with cold pain and precold baseline. Thus, to examine the sources of theta power increase during chronic and acute pain relief, source estimation was calculated. Chronic pain relief compared with baseline pain was associated with significant foci of theta power increase (Fig. 7A, left panels). Those foci were located in the left lateral prefrontal cortex (left DLPFC, Montreal Neurological Institute (MNI) coordinates: −46, 38, 8) and midline frontal cortex (left hemisphere, MNI: −9, 41, 10; right hemisphere, MNI: 15, 40, 10), all *P*s < 0.05. Compared with baseline, acute pain relief resulted in robust theta power increase in several pain-related areas, such as the left DLPFC (MNI: −46, 38, 8) and midline frontal cortex (left hemisphere, MNI: −1, 27, 19; right hemisphere, MNI: 3, 28, 19), all *P*'s < 0.05 (Fig. 7A, right panels). Chronic pain relief vs acute pain relief comparison revealed a significantly larger theta power increase in the midline frontal cortex (left hemisphere, MNI: −1, 27, 19; right hemisphere, MNI: 3, 28, 19), both *P*s < 0.05 (Fig. 7B).

## 4. Discussion

As approaches to treat pain evolve, deeper insight into the associations between cortical activity and pain relief are essential. Biomarkers associated with pain reduction have the potential to better guide therapies and improve the development of future analgesic interventions. Although several studies have addressed oscillatory brain activity during pain, the electrophysiological signatures of pain relief are far less explored. This is most notably true for chronic pain. Thus, reduction in pain intensity after the nerve block procedure in chronic pain patients provided a unique model to identify clinically relevant cortical dynamics with good temporal precision. To further refine how the cortical physiology of relief from *chronic* pain is distinct from that of reduction in acute experimental pain, we used data from healthy participants undergoing cold-pressor test to serve as a control group. The novel finding of this study is that reduction in both chronic clinical pain and acute experimental pain was associated with significant theta power increase at the frontal area. Importantly, midfrontal theta power rebound during chronic pain relief showed significant positive correlation with the magnitude of pain reduction after the nerve block procedure. Furthermore, this study for the first time compared cortical sources of chronic vs acute pain relief. Although both the left DLPFC and the midline frontal cortex showed theta power increase during chronic and acute pain relief, theta power in the midline frontal cortex was distinctly elevated with chronic pain relief. Taken together, these findings support that there are specific theta rhythm cortical dynamics from the medial frontal lobe associated with a meaningful relief of chronic pain.

Because frontal cortex is involved in the processing of painful sensations,^7,14,65^ it is possible that an increase in frontal theta power might be a consequence of nerve block-driven pain relief. Findings from neuromodulation studies have suggested that increases in activity in the prefrontal cortical areas are important for treating pain.^16,17,19^ This study implies that successful pain relief may be associated with increase in midfrontal theta activity. However, as mechanisms underlying cortical generation of theta power with regard to pain relief are limited, understanding the neural circuits defining this active phenomenon of pain relief will require further study.

Beyond the more dynamic alterations associated with active pain relief, there is also a question of how patients with chronic pain differ from healthy participants in their baseline physiology. Compared with healthy controls, there was a tendency toward increased delta and theta power in patients with chronic pain. Literature on the baseline electrophysiology between patients with and without pain is not consistent (see Refs. 52 and 53 for a review). One noticed abnormality is an increase of theta oscillations in chronic pain patients.^4,62,67^ Sarnthein et al. proposed that this finding may reflect TCD, which provides a theta pacing mechanism that acts to perpetuate pain. However, conflicting evidence suggests that thalamic bursts may be positively^24,33,37,39^ or negatively correlated with pain.^12,29,30,54^ Our sample was likely too small to detect significant differences in the relevant baseline EEG parameters between chronic pain patients and healthy controls, and it was not an objective of the study. However, there were studies that did not observe abnormal baseline theta oscillations in chronic pain patients.^35,63^ In a previous studies involving chronic pain patients, which reported statistically significant theta overactivity at baseline, ie, TCD, patients had much stronger pain compared with moderate pain in our study. This implies that only very strong pain can elicit TCD. The inconsistency of findings with regard to TCD may be further related to the difference in pain medications usage rates across studies.

Theta rhythms can play different roles in pain processing. To better spatially characterize chronic pain relief, we estimated the cortical source generators of pain relief. Chronic and acute pain relief were both characterized by significant theta power rebound at the frontal area with its sources located in the left DLPFC and midline frontal cortex. Previous studies support critical involvement of the prefrontal and cingulate areas in the cortical processing of long-lasting acute painful stimuli.^36,43,50,64,72^ Notably in this study, although both acute and chronic pain relief were associated with theta power increases that localized to the left DLPFC and midline frontal cortex, chronic pain relief had a larger rebound in the midline frontal cortex. Although a limited spatial resolution may not allow to pinpoint the specific brain area, we think the data point toward the involvement of the anterior cingulate cortex in chronic pain relief. This is in line with previous studies that have shown that chronic pain particularly engages the medial prefrontal cortex and anterior cingulate cortex.^1,2,25^ These results suggest that resolution of chronic pain may be more related to involvement of areas encoding to emotional–motivational processes, whereas the resolution of acute pain may be more associated with changes in sensorimotor areas.^50,64,72^

The pattern of EEG activity reported by previous investigators in healthy participants experiencing experimental long-lasting acute pain has largely been replicated in this study. The cold stimulation resulted in moderate acute pain leading to decrease of delta, theta, alpha, and beta powers. Decrease in delta and beta frequency bands showed lateralization, possibly because of the involvement of the contralateral somatosensory area for the hand. These findings are in line with previous studies on tonic pain processing.^6,8,10,11,13,18,21,22,28,44,66^ Alpha power reduction during painful stimulation is a well-known phenomenon possibly related to nonspecific arousal and attention to pain.^11,51^ Thus, the alpha suppression is unlikely to be a pain-specific phenomenon.^5,9^ The decrease of theta, delta, and beta powers in centro-parietal area may be related to the activation of nociception under the painful stimulation, although the specificity of these findings remains unclear. With regard to the gamma power, a number of studies have shown increased gamma oscillations during phasic or tonic pain.^18,46,49,57,64^ Prefrontal gamma synchronization in our study may represent increased top-down cognitive control to suppress pain experience.

## 5. Limitations

This study has the following limitations. Our sample size was limited to 12 participants per group. There were differences between the patient and control groups regarding sex and pain location. Patients had different comorbidities and they were on a variety of pain medications that may have introduced the potential confounders. Additionally, our source estimation results should be interpreted with caution considering a limited spatial resolution of low-density EEG.

## 6. Conclusion

In summary, our findings demonstrate that theta power rebound in the midline frontal cortex is associated with chronic pain relief. Through techniques as neurofeedback and neuromodulation, those features could be used for closed loop systems to modify a patient's pain experience. Studies exploring such interventions are warranted.

## Disclosures

E.C. Leuthardt has stock ownership in Neurolutions, Inner Cosmos, and Sora Neuroscience. Washington University also owns stock in Neurolutions. This work and E.C. Leuthardt have had their conflict of interest rigorously evaluated and managed throughout this study and with creation of this manuscript. The remaining authors have no conflicts of interest to declare.
